# Supplementary material for: Identifying SARS-CoV-2 Variants of Concern through Saliva-Based RT-qPCR by Targeting Recurrent Mutation Sites
Source: Microbiol Spectr. 2022 May 12;10(3):e00797-22. doi: 10.1128/spectrum.00797-22 (PMC9241879; doi:10.1128/spectrum.00797-22)
Supplement: SUPPLEMENTAL FILE 7 — Supplemental material. Download spectrum.00797-22-s007.pdf, PDF file, 0.4 MB [file spectrum.00797-22-s007.pdf]

Supplemental Materials FOR Publication

Supplemental Table 1. Reaction efficiencies for all primer and probe sets in multiplex.

RT-qPCR Assay	Multiplex Reaction Efficiency		
	Internal Control Probe	Reference Probe	Deletion/Substitution Probe
SΔ69-70	95.49%	100.57%	97.12%
ORF1a Δ3675-3677	101.56%	95.63%	101.44%
K417T	N/A	101.97%	89.52%
E484K	N/A	99.04%	102.19%
E484Q	N/A	94.69%	101.40%
L452R	N/A	103.99%	112.04%

Supplemental Table 2. Thermocycling Conditions for RT-qPCR assays.

SΔ69-70 and ORF1aΔ3675-3677			
**Stage**	**Temperature (°C)**	**Duration**	**Cycles**
Warm Up	25.0	2 min	1
Reverse Transcription	50.0	15 min	1
Initial Denaturation	95.0	2 min	1
Touchdown	95.0	3 sec	3
	72.0	30 sec	
	95.0	3 sec	3
	68.0	30 sec	
	95.0	3 sec	3
	64.0	30 sec	
Main Amplification	95.0	3 sec	45
	60.0	30 sec	
TaqPath SNP Assays (K417T, E484K, E484Q, and L452R)			
**Stage**	**Temperature (°C)**	**Duration**	**Cycles**
Warm Up	25.0	2 min	1
Reverse Transcription	50.0	15 min	1
Initial Denaturation	95.0	2 min	1
Main Amplification	95.0	3 sec	45
	60.0	30 sec	

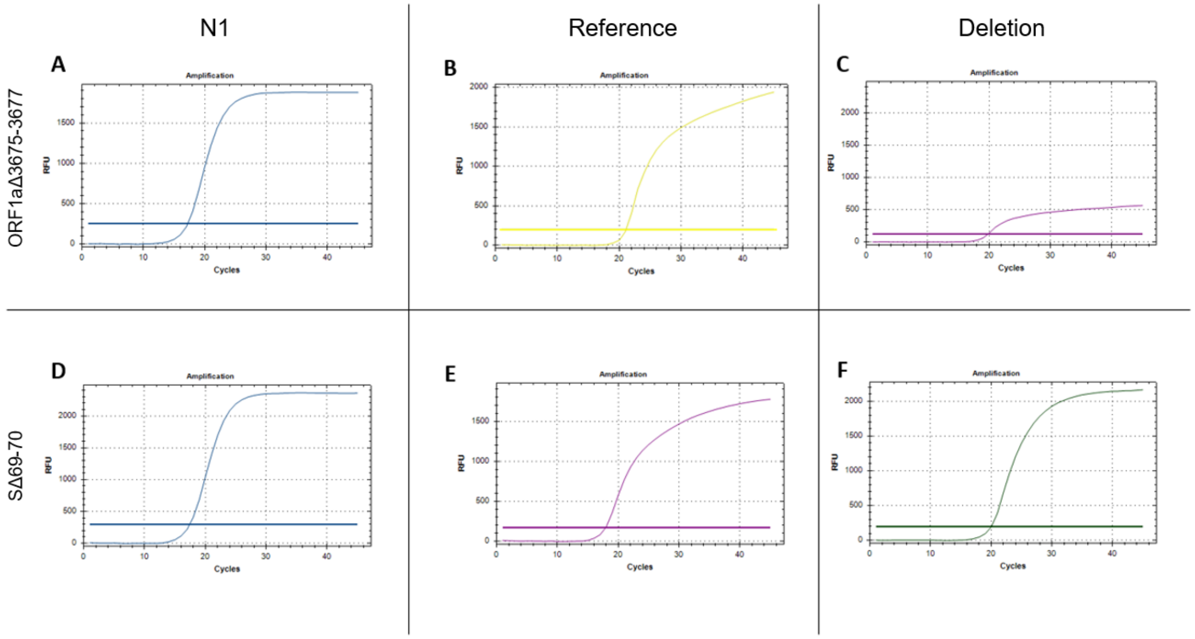

Supplemental Figure 1. Representative curves of RT-qPCR deletion assays.

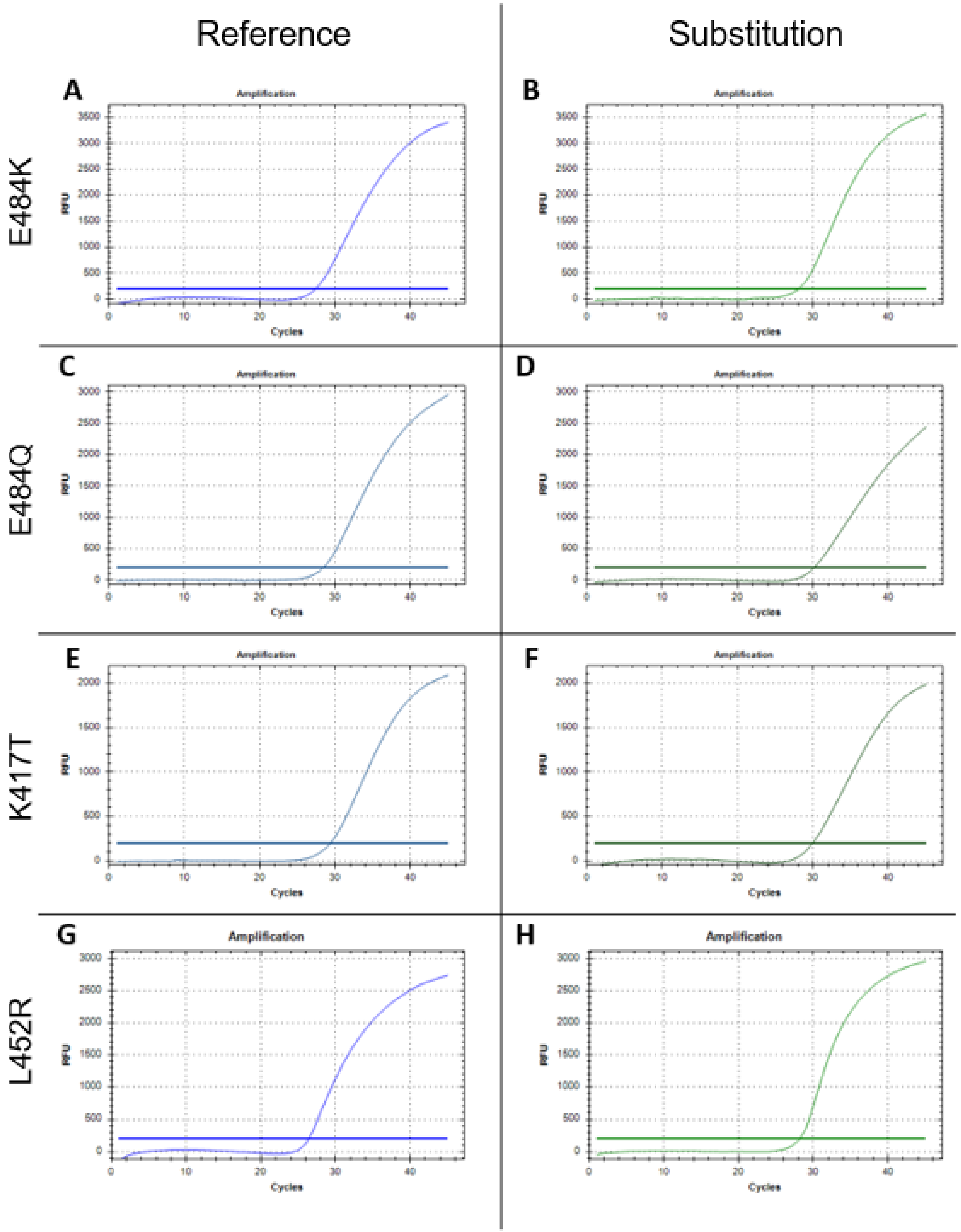

Supplemental Figure 2. Representative curves of TaqPath Spike SNP assays.

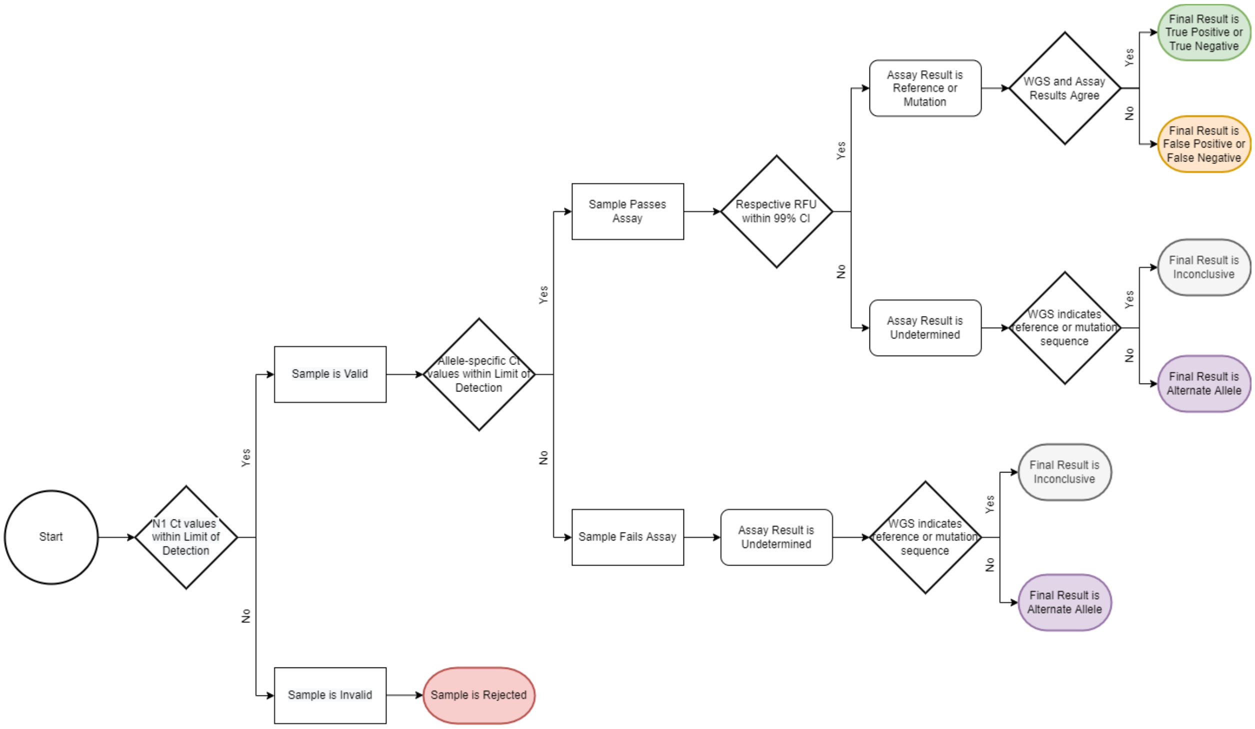

Supplemental Figure 3. Sample Resulting Flowchart.

Supplemental File 1. Standard Curve Analysis of Deletion and TaqPath Spike SNP Assays

Supplemental File 2. Master Data Sheet

Supplemental File 3. Sequenced Sample List with GenBank and GISAID Information

Supplemental File 4. Deletion Assay Resulting Sheet

Supplemental File 5. SNP Assay Resulting Sheet

Supplemental File 6. Omicron Screening Results
